# Effects of Atorvastatin Dose and Concomitant Use of Angiotensin-Converting Enzyme Inhibitors on Renal Function Changes over Time in Patients with Stable Coronary Artery Disease: A Prospective Observational Study

**DOI:** 10.3390/ijms17020106

**Published:** 2016-02-02

**Authors:** Ewa Wieczorek-Surdacka, Jolanta Świerszcz, Andrzej Surdacki

**Affiliations:** 1 Department of Nephrology, University Hospital, 31-501 Cracow, Poland; 2 Second Department of Cardiology, Jagiellonian University Medical College and University Hospital, 31-501 Cracow, Poland; grasshoppers@interia.eu (J.S.); surdacki.andreas@gmx.net (A.S.)

**Keywords:** angiotensin-converting enzyme inhibitors, coronary artery disease, glomerular filtration rate, renal function decline, statins

## Abstract

Angiotensin-converting enzyme inhibitors (ACEI) and statins are widely used in patients with coronary artery disease (CAD). Our aim was to compare changes in glomerular filtration rate (GFR) over time in subjects with stable CAD according to atorvastatin dose and concomitant use of ACEI. We studied 78 men with stable CAD referred for an elective coronary angiography who attained the then-current guideline-recommended target level of low-density lipoproteins (LDL) cholesterol below 2.5 mmol/L in a routine fasting lipid panel on admission and were receiving atorvastatin at a daily dose of 10–40 mg for ≥3 months preceding the index hospitalization. Due to an observational study design, atorvastatin dosage was not intentionally modified for other reasons. GFR was estimated during index hospitalization and at about one year after discharge from our center. Irrespective of ACEI use, a prevention of kidney function loss was observed only in those treated with the highest atorvastatin dose. In 38 subjects on ACEI, both of the higher atorvastatin doses were associated with increasing beneficial effects on GFR changes (mean ± SEM: −4.2 ± 2.4, 1.1 ± 1.6, 5.2 ± 2.4 mL/min per 1.73 m^2^ for the 10-mg, 20-mg and 40-mg atorvastatin group, respectively, *p* = 0.02 by ANOVA; Spearman’s *rho* = 0.50, *p* = 0.001 for trend). In sharp contrast, in 40 patients without ACEI, no significant trend effect was observed across increasing atorvastatin dosage (respective GFR changes: −1.3 ± 1.0, −4.7 ± 2.1, 4.8 ± 3.6 mL/min per 1.73 m^2^, *p* = 0.02 by ANOVA; *rho* = 0.08, *p* = 0.6 for trend). The results were substantially unchanged after adjustment for baseline GFR or time-dependent variations of LDL cholesterol. Thus, concomitant ACEI use appears to facilitate the ability of increasing atorvastatin doses to beneficially modulate time-dependent changes in GFR in men with stable CAD.

## 1. Introduction

As early as 2001, a meta-analysis of randomized controlled studies by Fried *et al.* [1] suggested the ability of 3-hydroxy-3-methylglutaryl-coenzyme A (HMG-CoA) reductase inhibitors (statins), to slow down renal function decline in patients with renal diseases. Five years later, a meta-analysis by Sandhu *et al.* [2] also identified renal benefits of statins, including a small yet significant reduction in the rate of kidney function loss, especially in populations with atherosclerotic cardiovascular (CV) disease. However, according to some recent meta-analyses, despite a lower risk of CV events in predialysis chronic kidney disease (CKD) subjects on statins, renoprotective statin effects were unclear with a significant heterogeneity across the included CKD studies [3,4]. Nikolic *et al*. [5] have recently reported a beneficial effect of statins on glomerular filtration rate (GFR) changes only for between one and three years of statin therapy in CKD. In contrast to these inconsistent results, a meta-analysis of controlled studies in CKD patients treated with atorvastatin revealed an evidence of the atorvastatin-induced renoprotection that was independent of follow-up duration and persisted after exclusion of any single trial from the analysis including the largest Treating to New Targets (TNT) study [6]. These discrepancies could be partially linked to a dosage-dependence of renal benefits of statins, recently demonstrated irrespective of the presence of CKD [7,8].

Angiotensin-converting enzyme inhibitors (ACEI) are recommended in CKD due to their well-recognized ability to reduce proteinuria and delay renal function decline. In addition, ACEI should be considered for CV event prevention even in patients with stable angina and without coexistent strong indications for this class of drugs, *i.e.*, left ventricular systolic dysfunction, diabetes or hypertension [9,10]. However, ACEI were used by only about 28% of the subjects participating in the TNT trial that demonstrated greater improvements in GFR over five years in patients with stable coronary artery disease (CAD) randomized to 80-mg atorvastatin *vs.* 10-mg atorvastatin [11]. Of note, in the Protection Against Nephropathy in Diabetes with Atorvastatin (PANDA) trial two-year GFR changes were similar in type 2 diabetics with early nephropathy allocated 80-mg and 10-mg atorvastatin, all of whom were receiving either ACEI or angiotensin II type 1 receptor blockers (ARB) [12].

Therefore, we hypothesized that dose-dependent effects of atorvastatin on renal function might be modified by ACEI use. Thus, the aim of our observational study was to compare GFR changes over time in men with stable CAD according to the dose of atorvastatin and concomitant usage of ACEI.

## 2. Results

Clinical and biochemical characteristics of our patients according to atorvastatin dose have been shown in Table 1.

**Table 1:** Patients’ characteristics according to atorvastatin dose.

Characteristic	Atorvastatin Daily Dose			*p*-Value
	10-mg, *n* = 29	20-mg, *n* = 24	40-mg, *n* = 25	
Age (years)	56 ± 10	57 ± 9	55 ± 10	NS
Hypertension, *n* (%)	25 (86%)	19 (79%)	20 (80%)	NS
Diabetes, *n* (%)	9 (31%)	6 (25%)	5 (20%)	NS
Left ventricular ejection fraction (%)	68 ± 6	70 ± 7	70 ± 6	NS
Body-mass index (kg/m^2^)	27.4 ± 3.4	26.8 ± 2.9	27.0 ± 3.1	NS
Mean blood pressure (mmHg)	99 ± 9	97 ± 9	96 ± 8	NS
LDL cholesterol (mmol/L)	2.1 ± 0.3	2.0 ± 0.4	2.2 ± 0.3	NS
HDL cholesterol (mmol/L)	0.9 ± 0.3	1.1 ± 0.3	1.0 ± 0.3	NS
Triglycerides (mmol/L)	1.7 ± 0.7	1.6 ± 0.6	1.5 ± 0.6	NS

Data are shown as mean ± SD; *p*-values by one-way ANOVA or chi-squared test. HDL: high-density lipoproteins; LDL: low-density lipoproteins; NS: non-significant.

Baseline GFR did not differ significantly across subgroups of patients treated with different atorvastatin doses (mean ± SEM: 52.4 ± 2.6, 59.3 ± 3.5, 62.5 ± 3.1 mL/min per 1.73 m^2^ for the 10-mg, 20-mg and 40-mg atorvastatin group, respectively; *p* = 0.4 by one-way analysis of variance [ANOVA]).

Out of 78 study subjects, 40 patients were receiving no ACEI, largely due to a history of adverse renal side-effects, which resulted in a significantly higher GFR in those with ACEI (69.5 ± 2.7 *vs.* 43.5 ± 2.0 mL/min per 1.73 m^2^, *p* < 0.001). Among ACEI users, study subjects were treated mainly with ramipril (2.5–10 mg daily), perindopril (2–8 mg daily) or enalapril (5–30 mg daily).

**Figure 1:**
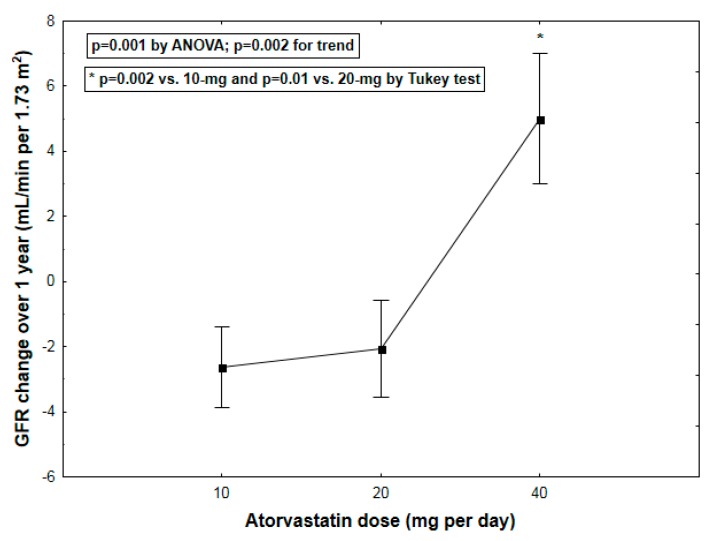
Changes of glomerular filtration rate (GFR) over 1 year (mean ± SEM) according to atorvastatin dosage.

Pooling all study subjects together, we found a significant effect of atorvastatin dose on changes in GFR, predominantly secondary to a GFR increase in those receiving 40-mg atorvastatin (GFR change: −2.6 ± 1.2, −2.1 ± 1.5, 5.0 ± 2.0 mL/min per 1.73 m^2^ for the 10-mg, 20-mg and 40-mg atorvastatin group, respectively, *p* = 0.001 by ANOVA; *p* = 0.002 for trend) (Figure 1).

ACEI use was not significantly associated with GFR changes (0.9 ± 1.4 *vs.* −0.5 ± 1.3 mL/min per 1.73 m^2^ for patients with and without ACEI, respectively, *p* = 0.6 by ANOVA).

Changes of GFR over one year did not correlate to any of the baseline clinical and biochemical characteristics (*p* > 0.1) or longitudinal changes in low-density lipoproteins (LDL) cholesterol (*p* = 0.4) and mean blood pressure (*p* = 0.5).

**Figure 2:**
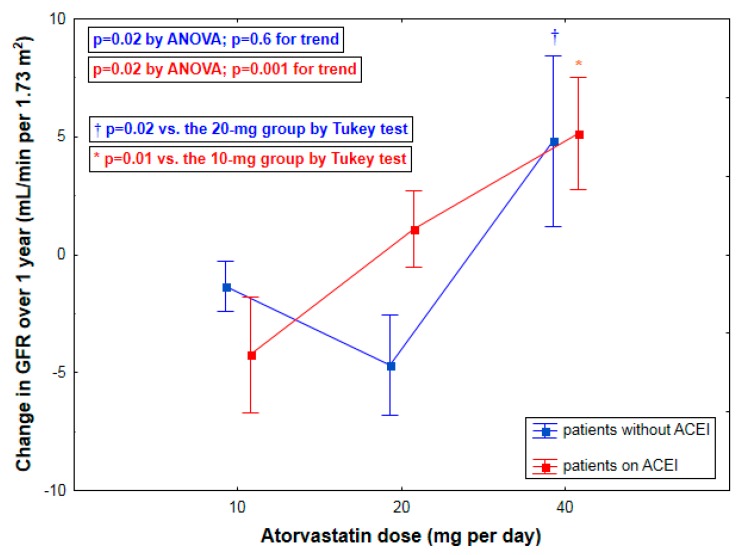
Changes of glomerular filtration rate (GFR) over one year (mean ± SEM) by atorvastatin dosage stratified by the use of angiotensin-converting enzyme inhibitors (ACEI).

Irrespective of ACEI use, a prevention of kidney function loss was observed only in those treated with the highest atorvastatin dose (Table 2; Figure 2). In subjects on ACEI (*n* = 38), both of the higher atorvastatin doses were accompanied by increasing beneficial effects on time-dependent changes in GFR (*p* = 0.02 by ANOVA; *rho* = 0.50, *p* = 0.001 for trend) (Table 2; Figure 2). In sharp contrast, in patients without ACEI (*n* = 40), no significant trend effect was observed (*p* = 0.02 by ANOVA; *rho* = 0.08, *p* = 0.6 for trend) (Table 2; Figure 2). With regard to the strength of these trend effects on longitudinal GFR changes across increasing atorvastatin dosage, a positive association in subjects on ACEI (*rho* = 0.50) differed significantly (*p* = 0.045) from the lack of respective relationship (*rho* = 0.08) in patients without ACEI.

**Table 2:** Glomerular filtration rate (GFR) by atorvastatin dose according to the use of angiotensin-converting enzyme inhibitors (ACEI).

GFR (mL/min per 1.73 m^2^)	Atorvastatin Daily Dose			*p*-Value by ANOVA	*p*-Value for Trend
	10-mg	20-mg	40-mg		
Patients on ACEI (*n* = 38)					
	*n* = 13	*n* = 11	*n* = 14		
Baseline	65.9 ± 4.8	74.7 ± 6.2	69.0 ± 4.0	NS	NS
Change over 1 year	−4.2 ± 2.4	1.1 ± 1.6	5.2 ± 2.4 *	0.02	0.001
Patients without ACEI (*n* = 40)					
	*n* = 16	*n* = 13	*n* = 11		
Baseline	41.4 ± 4.5	49.3 ± 5.3	41.3 ± 4.3	NS	NS
Change over 1 year	−1.3 ± 1.0	−4.7 ± 2.1	4.8 ± 3.6 ^†^	0.02	NS

Data are shown as mean ± SEM; *p*-values by one-way ANOVA. * *p* = 0.01 *vs.* the 10-mg group; ^†^ *p* = 0.02 *vs.* the 20-mg group by Tukey’s test; NS: non-significant.

By multivariate analysis, a change in GFR was positively related to the treatment with 40-mg atorvastatin. In addition, we found a significant interaction between ACEI use and the therapy with 20-mg atorvastatin (Table 3). Accordingly, in patients with ACEI we observed a higher GFR change in the 20-mg atorvastatin group compared to the 10-mg atorvastatin group, whereas in those not receiving ACEI an increase in GFR was found exclusively for the 40-mg group (Figure 2). The results were substantially unchanged after inclusion of baseline GFR or time-dependent variations of LDL cholesterol and mean blood pressure into the model.

**Table 3:** Multiple linear regression analysis of predictors a change in GFR over 1 year.

Predictor	Regression Coefficient		*p*-Value
	Mean ± SEM	95% CI	
Atorvastatin daily dose			
40-mg *vs.* 10-mg	3.9 ± 1.1	1.8–6.0	<0.001
20-mg *vs.* 10-mg	0.5 ± 1.1	−1.7–2.7	0.7
Treatment with ACEI (yes *vs.* no)	1.5 ± 1.2	−0.7–3.8	0.2
Interactions of ACEI use and atorvastatin dose			
ACEI use and 20-mg atorvastatin	2.3 ± 1.1	0.1–4.5	0.04
ACEI use and 40-mg atorvastatin	0.8 ± 1.1	−1.3–2.9	0.5

*R*^2^ for the whole model: 0.22; *p* = 0.003.

## 3. Discussion

Our salient finding was the ability of ACEI use to modulate the relation between atorvastatin dose of 10–40 mg daily and GFR changes over a mean follow-up of 12 months in men with stable CAD. In the study subjects on ACEI, the use of atorvastatin was associated with increasing beneficial effects on kidney function beginning from an atorvastatin dose of 20 mg, whereas in those receiving no ACEI, no significant trend effect was found. Additionally, time-dependent changes in GFR appeared independent of concomitant longitudinal variations of LDL cholesterol or blood pressure.

### 3.1. Mechanistic Considerations

Our findings may be indicative of the relevance of cholesterol-independent pleiotropic statin actions for the renal benefit achieved with atorvastatin. Admittedly, cholesterol lowering through HMG-CoA reductase inhibition is the principal mechanism of statin actions; however, because cholesterol levels were similar in patients treated with three atorvastatin doses, a hypothetical role of cholesterol-independent pathways can be hypothesized. Beyond cholesterol reduction, pleiotropic statin effects include attenuation of endothelial dysfunction as well as anti-inflammatory, antioxidant, antiproliferative, antifibrotic and sympatholytic activity [5,13,14], all of which were also shown for ACEI [15]. Moreover, adding a statin to ACEI ameliorated renal function, reduced proteinuria and prevented glomerulosclerosis, tubular damage and interstitial fibrosis in several experimental models of kidney disease [16,17,18,19]. Additionally, statins are able to down-regulate angiotensin II type 1 receptors [20,21], whose density is known to increase in patients on ACEI. Thus, it does not seem implausible to assume that converging intercellular pathways mediating ACEI and statin activities could be responsible for the association of favorable GFR changes with statin therapy at a lower dose of atorvastatin in ACEI users compared with those receiving no ACEI. This concept appears much more likely than altered atorvastatin metabolism due to a difference in renal function according to ACEI use because the drug is eliminated primarily in the bile and no dose adjustment is required in CKD [22].

Admittedly, it is doubtful whether long-term effects of statin therapy on kidney structure in animal models of CKD [16,17,18,19] could translate into measurable effects on GFR within a relatively short follow-up in the present study. Therefore, we can hypothesize that our findings might rather be linked to hemodynamic statin actions. This concept was also proposed by Athyros *et al.* [23] as an explanation for early increase in GFR already at the sixth week of treatment with the starting atorvastatin dose of 10 mg/day in subjects with established CAD and dyslipidemia participating in the GREek Atorvastatin and Coronary heart disease Evaluation (GREACE) study, with the greatest benefit in those with early renal dysfunction. It is noteworthy that ACEI—shown to delay CKD progression on a long-term basis [15]—did not independently influence GFR changes in the present study.

Statins may exert renal hemodynamics via preglomerular vasodilation [24], a likely consequence of an increased bioavailability of nitric oxide (NO), all the more because short-term (six weeks) treatment with rosuvastatin potentiated the dependence of renal plasma flow on NO generation in patients with hypercholesterolemia, with a trend towards improved basal NO activity already after three-day therapy [25]. Of note, in healthy subjects the dependence of fractional sodium excretion on the l-arginine–NO pathway was more pronounced after five-day treatment with 80 mg atorvastatin daily [26]. In addition, GFR improved after a four-week therapy with 40 mg simvastatin per day in patients with autosomal dominant polycystic kidney disease, which was associated with improved endothelium-dependent dilation in the forearm [27]. On the other hand, in subjects with type 2 diabetes, administration of atorvastatin (10 or 80 mg/day) for 30 weeks neither improved impaired flow-mediated dilation of the brachial artery [28] nor affected urinary markers of systemic NO generation and oxidative stress [29] as compared to patients randomized to placebo.

Elevated bioavailability of renal NO produces not only the dilation of glomerular arterioles, but also local NO generated in the juxtaglomerular apparatus can antagonize the angiotensin II-dependent enhancement of vasoconstrictory responses of afferent arterioles via tubuloglomerular feedback (TGF) [30]. In keeping with this concept, Song *et al.* [31] have recently demonstrated augmented TGF responsiveness associated with increased superoxide generation and decreased NO formation by neuronal NO synthase in the *macula densa* in a model of hypertension induced with slow-pressor infusion of angiotensin II in mice. Furthermore, Park *et al.* [32] observed the ability of rosuvastatin to protect against angiotensin II-induced inflammatory and profibrotic activation and renal injury in a transgenic model of angiotensin II-induced hypertension and target-organ damage. Finally, atorvastatin attenuated renal oxidative stress, exerted anti-inflammatory and antifibrotic effects, prevented glomerulosclerosis and upregulated renal endothelial NO synthase in a rat model of salt-sensitive hypertension associated with increased angiotensin II-dependent activation of nicotinamide adenine dinucleotide phosphate oxidases [33].

Thus, we can hypothesize that restoration of renal NO activity on statins could be especially beneficial under ACE inhibition because it might possibly maintain an equilibrium between the angiotensin II-mediated postglomerular vasoconstriction and the TGF-dependent preglomerular vasoconstriction, thereby preventing excessive GFR reductions on ACEI.

### 3.2. Clinical Implications

Irrespective of these speculative mechanistic considerations, the results of the present study may translate into bedside, although the study population, with a prevalence of diabetes of only 25%, could be considered at low risk for CKD progression upon exclusion of subjects with heart failure, left ventricular systolic dysfunction and uncontrolled hypertension. In the clinical practice, ACEI and ARB tend to be underused in patients with increasing stages of CKD due to perceived safety concerns about adverse renal effects of these drugs [34], which was also evident in our study group. Therefore, subjects with CKD, who are receiving no ACEI therapy, are devoid of a valuable tool in the medical armamentarium aimed at slowing down CKD progression, being thus more dependent on alternative strategies to protect against GFR deterioration. Accordingly, since we were able to detect the ability of 40-mg atorvastatin to prevent a fall of GFR over a one-year follow-up period in ACEI non-users, higher atorvastatin doses may be a reasonable option in this clinical setting not only to attenuate the risk of adverse CV outcome but also to prevent renal function decline.

Furthermore, the ability of statin to improve GFR appears augmented in CKD because, in the TNT study, the mean percentage increase from baseline GFR was higher among participants with a baseline GFR below 60 mL/min per 1.73 m^2^ both for the patients allocated 80-mg atorvastatin (9.9% *vs.* 7.6%) and in the 10-mg arm (6.6% *vs.* 5.2%) [11]. In addition, relative GFR improvements associated with titrate-to-goal regimens of atorvastatin (10 to 80 mg per day) over a regimen of “usual care” were more pronounced in individuals with established CAD and coexistent CKD compared to those with a normal renal function in the GREACE (15% [GFR < 77 mL/min] *vs.* 3% [GFR > 77 mL/min]; mean atorvastatin dose: 24 mg/day) [23] and Aggressive Lipid-Lowering Initiation Abates New Cardiac Events (ALLIANCE) trial (4.5% [CKD] *vs.* 1.1% [no CKD]; mean atorvastatin dose: 40.5 mg/day) [35].

In some analogy to these reports, in the patients treated with 40-mg atorvastatin, we observed a higher relative increase in GFR in those without ACEI (about 12%) *vs.* ACEI users (about 7.5%), whereas average GFR was lower by almost 30 mL/min per 1.73 m^2^ in the former. However, GFR changes according to ACEI/statin use were previously reported only in a *post hoc* analysis of the GREACE study, being similar during a three-year follow-up in patients taking the combination of a statin (mainly atorvastatin) plus an ACEI and those receiving a statin without ACEI [36]. With regard to the TNT [11] and ALLIANCE [35] study, renal effects of atorvastatin according to the treatment with ACEI/ARB have not been reported so far.

It needs to be acknowledged that we were not able to make comparisons with the reference to patients without statins. However, assuming the notion of the hypothetical ACEI–statins interactions in terms of GFR changes, the magnitude of GFR improvements over time can possibly be perceived as a renal “mirror” of additive beneficial effects of ACEI and statins on clinical outcome, previously demonstrated in patients with CAD [36] and recently in diabetic subjects with critical limb ischemia [37]. Moreover, the proposed concept can also indirectly imply a higher ability of lower atorvastatin doses to produce beneficial effects on renal function in ACEI users *vs.* non-users, which was found in the present study. Of note, in a placebo-controlled study, Bianchi *et al.* [38] observed that adding a mean dose of 15 mg of atorvastatin daily to ACEI or ARB prevented one-year renal function decline and reduced proteinuria in patients with mild-to-moderate CKD due to idiopathic chronic glomerulonephritis. Unfortunately, the proportional use of ACEI and ARB was not reported by the Lipid Lowering and Onset of Renal Disease (LORD) trial investigators who observed no statistical difference in 2.5-year GFR changes between CKD subjects of various etiology randomized to 10-mg atorvastatin and placebo [39].

### 3.3. Study Limitations

First, a relatively low number of the patients and observational study design are major study limitations and a randomized assignment of the subjects to ACEI therapy and different atorvastatin doses would be more appropriate. However, we analyzed only patients who were already on atorvastatin (with or without ACEI) for at least three months preceding the index hospitalization and attained the then-current guideline-recommended target levels of LDL cholesterol in a routine fasting lipid panel on admission; Second, we did not routinely estimate proteinuria and indices of calcium/phosphorus metabolism, also known to affect CKD progression; Finally, because study subjects were followed on an outpatient basis, we were able to collect exclusively a self-reported history of medication use after discharge from our department.

## 4. Experimental Section

### 4.1. Patients

We studied men with stable CAD referred for an elective diagnostic coronary angiography who exhibited the presence of at least one epicardial coronary narrowing of ≥70% and were being treated in accordance with current practice guidelines including aspirin and statin for ≥3 preceding months. All patients underwent a complex percutaneous coronary angioplasty in our center. As described previously, a wide set of exclusion criteria was applied to reduce the heterogeneity of the study population [40]. We had *a priori* excluded subjects with heart failure/left ventricular systolic dysfunction, significant valvular heart disease or congenital heart disease, arterial hypertension not adequately controlled by drugs, a history of acute coronary syndrome within the previous three months, major surgery in the past six months, GFR >120 or <30 mL/min per 1.73 m^2^ of body-surface area, evidence of abnormal liver function, contrast nephropathy during index hospitalization, relevant abnormalities in routine blood analysis, coexistent inflammatory or malignant diseases, and chronic medication with non-steroidal anti-inflammatory drugs or coxibs. In the present study, we analyzed only subjects who attained the then-current guideline-recommended target level of LDL cholesterol below 2.5 mmol/L [9,10] in a routine fasting lipid panel on admission and were receiving daily atorvastatin doses between 10 and 40 mg prior to the index hospitalization. Due to an observational study design, the doses of atorvastatin were not intentionally modified for other reasons in the analyzed patients. Out of 391 pre-screened patients, 282 were eliminated from the study on the basis of the above listed inclusion and exclusion criteria.

The study was approved by the ethical committee of our institution as indicated previously [40,41] and the patients gave informed consent.

### 4.2. Collection of Follow-up Data

After discharge from our department, patients underwent routine control visits in our outpatient clinic [41] and, if deemed necessary from clinical practice indications, re-hospitalizations in our center. In order to assess GFR changes over about a one-year follow-up, we recorded GFR—estimated by the Chronic Kidney Disease Epidemiology Collaboration formula [42]—during the index hospitalization and at 12 ± 3 months after discharge.

We excluded from the study the patients in whom atorvastatin doses were changed and/or ACEI were permanently discontinued or introduced by an attending physician before the control visit. Among 109 eligible patients who were followed, 31 were eliminated from the final analysis because of a modification of the therapeutic regimen with regard to atorvastatin/ACEI after the index hospitalization, or due to loss to follow-up.

### 4.3. Statistical Analysis

Data are presented as means and SD or SEM for continuous variables or *n* (%) for categorical characteristics. In order to check univariate effects of atorvastatin dose and ACEI use on changes in GFR, ANOVA was applied with either drug dosage or ACEI therapy as a categorical predictor. *Post hoc* comparisons were done by Tukey’s test. The ANOVA assumptions, *i.e.*, the accordance with a Gaussian distribution and uniformity of variance were tested by the Kolmogorov–Smirnov and Levene test, respectively. Pearson’s correlation coefficients (*r*) were computed to assess univariate associations between, on the one side, changes in GFR, and on the other side, baseline clinical and biochemical characteristics as well as time-dependent changes of LDL cholesterol and mean blood pressure.

For GFR changes over time, testing for trend across increasing doses of atorvastatin was performed with Spearman’s rank-order correlation coefficients (*rho*) in the study subjects stratified according to ACEI use. Then, *rho* coefficients between those with and without ACEI were compared using Fisher’s *r*-to-*z* transformation because Myers and Sirois [43] demonstrated that treating *rho* coefficients as if they were Pearson’s coefficients was more robust than alternative approaches with respect to false rejection of a true null hypothesis. A *p*-value below 0.05 was inferred significant.

Then, in order to check whether the effects of atorvastatin dosage on renal function were affected by ACEI therapy, a generalized linear regression model was applied with a change in GFR as a dependent variable. Since preliminary analyses did not allow the assumption of monotonic relations between atorvastatin dose and the dependent variable across subgroups of the study subjects divided on the basis of ACEI use, we entered 20-mg and 40-mg doses of atorvastatin as separate categorical predictors. Then, effects of the both statin doses, ACEI use and interactions between ACEI usage and each of the two statin doses were estimated. Regression coefficients for these factors are equivalent to an effect on GFR in the patients exposed to a factor of interest with the reference to the patients without this exposure. In order to adjust for potential continuous confounders, we planned to enter into the model baseline characteristics for which the univariate *p*-value did not exceed 0.10. Additionally, to control for time-dependent changes in LDL cholesterol and mean blood pressure, the analyses were repeated with these covariates in the regression model. We also compared results of the two regression models with and without inclusion of baseline GFR as a potential confounder. We used this approach because, on the one hand, patients without ACEI exhibited significantly lower GFR due to a history of adverse renal effects and, on the other hand, the adjustment for baseline levels of a variable may result in a measurement error-dependent bias when searching for determinants of changes in this variable over time [44,45].

## 5. Conclusions

Concomitant ACEI use appears to facilitate the ability of increasing atorvastatin doses to beneficially modulate time-dependent changes in GFR in men with stable CAD.

## Abbreviations

ACEI: angiotensin-converting enzyme inhibitors
ANOVA: analysis of variance
ARB: angiotensin II type 1 receptor blockers
CAD: coronary artery disease
CKD: chronic kidney disease
CV: cardiovascular
GFR: glomerular filtration rate
HDL: high-density lipoproteins
HMG-CoA: 3-hydroxy-3-methylglutaryl-coenzyme A
LDL: low-density lipoproteins
NO: nitric oxide
NS: non-significant
SEM: standard error of the mean
SD: standard deviation
TGF: tubuloglomerular feedback
